# Interaction between the hypothalamo-pituitary-adrenal and thyroid axes during immobilization stress

**DOI:** 10.3389/fphys.2022.972171

**Published:** 2022-10-18

**Authors:** Hakeem J. Kadhim, Wayne J. Kuenzel

**Affiliations:** ^1^ Veterinary Medicine College, University of Thi-Qar, Nasiriyah, Iraq; ^2^ Poultry Science, University of Arkansas, Fayetteville, AR, United States

**Keywords:** immobilization stress, NHpC, trh, TRHR1, CRH2, TSH, CRHR2

## Abstract

The location of corticotropin-releasing hormone receptor 2 (CRH-R2) on thyrotropes within the avian anterior pituitary (APit) and its activation by different stressors indicate a possible communication between hypothalamo-pituitary-adrenal (HPA) and thyroid (HPT) axes. Therefore, an experiment was designed to 1) compare the timing of major components of the HPT axis to those of the HPA axis; 2) address whether stressors activating the HPA axis may simultaneously upregulate components of the HPT axis. Blood, brain, and APit were sampled from chicks prior to stress (control) and 15, 30, 60, 90, and 120 min following immobilization (IM) stress. The nucleus of the hippocampal commissure (NHpC) and paraventricular nucleus (PVN) were cryo-dissected from brains for RT-qPCR. Gene expression of thyrotropin-releasing hormone (TRH) and its receptors (TRH-R1 and TRH-R3), urocortin3 (UCN3), deiodinase 2 (D2), and the second type of corticotropin-releasing hormone (CRH2) within the NHpC and PVN was measured. Additionally, gene expression of TRH receptors, thyroid stimulating hormone subunit beta (TSHβ), and D2 was determined in the APit and corticosterone assayed in blood. In brains, a significant upregulation in examined genes occurred at different times of IM. Specifically, UCN3 and CRH2 which have a high affinity to CRH-R2 showed a rapid increase in their mRNA levels that were accompanied by an early upregulation of TRHR1 in the NHpC. In the APit, a significant increase in gene expression of TSHβ and TRH receptors was observed. Therefore, results supported concurrent activation of major brain and APit genes associated with the HPA and HPT axes following IM. The initial neural gene expression originating within the NHpC resulted in the increase of TSHβ mRNA in the APit. Specifically, the rapid upregulation of UCN3 in the NHpC appeared responsible for the early activation of TSHβ in the APit. While sustaining TSHβ activation appeared to be due to both CRH2 and TRH. Therefore, data indicate that CRH-producing neurons and corticotropes as well as CRH- and TRH-producing neurons and thyrotropes are activated to produce the necessary energy required to maintain homeostasis in birds undergoing stress. Overall, data support the inclusion of the NHpC in the classical avian HPA axis and for the first time show the concurrent activation of the HPA axis and components of the HPT axis following a psychogenic stressor.

## 1 Introduction

In birds as in mammals, the HPA axis is essential and important for regulating stress. The hypothalamic PVN contains two peptides, CRH and arginine vasopressin/vasotocin (AVP/AVT), that drive the HPA axis. The two neuropeptides are transported to the APit and bind to G protein-coupled receptors located on the cell membrane of corticotropes to activate proopiomelanocortin (POMC) genes within the cells that ultimately produce an adrenocorticotropic hormone (ACTH) (Blas, 2015). Secretions of ACTH into the cardiovascular system are carried to the adrenal glands for synthesis and release of glucocorticoids [cortisol in humans and corticosterone (CORT) in birds and other vertebrates] to redirect energy resources to meet real or anticipated demands (Romero, 2004; Carsia, 2015; Herman and Tasker 2016).

The corticotropin-releasing hormone binds primarily to its receptor 1 (CRH-R1) in mammals and other vertebrates located on corticotropes (Aguilera et al., 2004). Less attention has been paid to the discovery of a second CRH receptor (CRH-R2) in birds located in APit thyrotropes (De Groef et al., 2004). Within the CRH family, the gene UCN3 was found to be expressed predominantly, but not exclusively in the hypothalamus, pons, and medulla of chick brains (Grommen et al., 2017). UCN3 modulates the behavioral and neuroendocrine system that helps maintain homeostasis in response to stress (Jamieson et al., 2006) and has a much higher affinity for CRH-R2 than CRHR-R1 (Lewis et al., 2001). Recently, a second CRH peptide was discovered, termed CRH2, and found across vertebrate classes (Grone and Maruska, 2015). The gene was identified in chickens (CRH2) and shown to be 15-fold more potent in activating CRH-R2 than CRH-R1 and potently stimulates TSHβ in pituitary cell culture studies (Bu et al., 2019). All brain regions showed high relative brain levels of CRH2 mRNA including the hypothalamus. Additionally, the pituitary gland showed the highest mRNA levels compared to the whole brain and 13 other tissues and organs sampled (Bu et al., 2019).

The presence of CRH-R2 on the thyrotropes indicates a direct positive or negative effect of CRH and/or CRH-like peptides (UCN3 and CRH2) on thyrotropes *via* CRH-R2. Ultimately, this could influence gene expression of TSHβ in addition to the effect of central secretion of TRH on the TSH secretion from APit *via* its receptors (TRH-R1 and TRH-R3). Hence, the net effects of CRH and CRH like peptides (CRH2 and UCN3) are to increase or decrease thyroid hormone (T4 and T3), indicating a possible interaction between the HPA and HPT axes, where the latter classically played an important role in growth, differentiation, and metabolism (De Groef et al., 2005; Brent, 2012; Mullur et al., 2014). However, the role of the HPT axis in regulating stress has received much less attention. Nonetheless, high concentrations of TRH have been shown to occur in the chicken hypothalamus and anterior pituitary (Geris et al., 1999) and TRH-R1 is expressed in the anterior pituitary (De Groef et al., 2003) and TRH-R3 is highly expressed in the hypothalamus and anterior pituitary (Li et al., 2020).

The distribution of CRH neurons is well characterized in the chicken brain (Richard et al., 2004). We found CRH neurons in a septal brain structure called the NHpC (Nagarajan et al., 2017a). Following stress, CRH neurons in the NHpC responded rapidly after initiation by two different stressors, feed deprivation (Nagarajan et al., 2017b; Kadhim et al., 2019) and immobilization (IM; Kadhim et al., 2021). The two stressors were used to determine the sequence of gene activation within the avian HPA axis. Since each of two different stressors activated CRH-R2 located on thyrotropes suggest strongly that stressors not only activate the HPA axis but also the HPT axis. We hypothesized that stress stimulates the HPA and components of the HPT axis, and both axes are activated concurrently. The following aims were addressed: 1) timing of neural secretion from the NHpC and PVN in TSHβ gene expression during stress; 2) gene expression of TRH, TRH-R1, and TRH-R3; 3) role of CRH2 and UCN3 in TSHβ gene activation; 4) possible role of the HPT axis in the neuroendocrine regulation of stress.

## 2 Materials and methods

### 2.1 Animals

Broiler chicks (one-day old male Cobb 500) were obtained from a commercial hatchery and raised on our poultry farm directly north of the University of Arkansas. Chicks were provided feed (a standard, broiler starter diet) and water *ad libitum* and exposed to continuous light for the first 3 days so that they could locate both the feed and water. Thereafter, birds were maintained under a daily photoperiod of 16 h: 8 h light/dark cycle (LD 16:8)T, lights on at 6:00 a.m. A heating program was started initially at 32°C and reduced by 2.5°C weekly until reaching 24°C. At 5 weeks of age, experiments were initiated, and sampling occurred between 8:00 a.m. and 4:00 p.m. Each experimental group of chicks had an equal representation of chicks sampled throughout the 8 h of daily sampling times by establishing four, consecutive 2 h time blocks. The same pattern of sampling was followed and repeated until 4:00 p.m. in order to minimize any potential diurnal variation on plasma samples analyzed for CORT levels by RIA (see Section 2.3). All procedures utilized (i.e., immobilization, housing conditions, handling, and sampling) were approved by the University of Arkansas Institutional Animal Care and Use Committee.

### 2.2 Stress procedure and sample collection for gene expression

Immobilization (IM) stress was initiated on week 5, with feed and water provided *ad libitum.* Chicks were randomly divided into six treatment groups (10–12 birds/group): one group was control, and the rest included five stressed groups: 15, 30, 60, 90, and 120 m following IM, where m = minutes of stress. In stressed groups, birds were secured in a harness where they could not move their wings nor stand, however, did have access to water and feed during the period of restraint. Stress treatments were designed so that all birds would be sampled individually directly after their stress period ended. First, blood was withdrawn from the brachial vein, transferred to a heparinized tube, and refrigerated. Each bird was then cervically dislocated and the brain and APit were rapidly dissected. Brain samples were immersed in two-methyl butane at −30°C for 15 s to maintain their structural morphology for cryo-sectioning and placed in dry ice before being stored at −80°C. Anterior pituitaries were likewise frozen in dry ice and stored at −80°C. Later, thick coronal sections of brain samples were cut at 100 µm using a cryostat and the targeted structures were punched with a stainless steel cannula (brain punch; Palkovits, 1973) using a glass pipette. The NHpC and PVN were micro-dissected, transferred to TRIzol, and stored at −20°C until processed for RNA extraction. Some samples were excluded due to insufficient RNA concentration. Final sample size per group was 8–10 birds/treatment. The procedural details were described in our recent publications (Nagarajan et al., 2017a; Nagarajan et al., 2017b; Kadhim et al., 2019; Kadhim et al., 2020; Kadhim et al., 2021).

### 2.3 Radioimmunoassay utilized for blood samples

Plasma was obtained from heparinized blood (*n* = 10–12/treatment) *via* centrifugation at 3,000 rpm for 20 m at 4°C and stored at −20°C until analysis of CORT concentrations by RIA (Proudman and Opel, 1989; Madison et al., 2008; Kadhim et al., 2021). Hemolyzed samples (1-2 samples/group) were excluded. Briefly, primary antibody (100 μl, polyclonal rabbit anti-CORT # 377, kindly provided by Dr. Proudman) and ^125^I corticosterone tracer (100 μl, MP Biomedicals Inc., Orangeburg, NY, US477A) were incubated with each sample for at least 24 h at 4°C. Sheep anti-rabbit antibody (200 μl) was used as the secondary antibody (MP Biomedicals Inc., Orangeburg, NY, United States). Counts/tubes were determined using a Perkin Elmer Wizard gamma counter. Samples were assayed in duplicate. After statistical analysis, data were expressed as the mean ± SEM for each group. In all studies, *p* < 0.05 was considered statistically significant. The intra-experimental coefficient of variance was less than 11%.

### 2.4 RNA extraction and purification, reverse transcription and qPCR procedure

Total RNA was extracted from frozen micro-dissected brain tissue and APit using Trizol-chloroform (Life Technologies) and described previously (Kadhim et al., 2020, 2019, 2021). Briefly, total RNA was extracted and treated with DNase I (Ambion, Austin, TX, United States) to eliminate genomic DNA followed by purification using an RNeasy mini kit (Qiagen). Total RNA concentration was estimated using Synergy HT multi-mode microplate reader (BioTek). Samples with an insufficient amount of RNA (1-3 samples) were excluded. First-strand cDNA was synthesized in 40 μl from total RNA (300 ng of NHpC, 500 ng of PVN, and 700 ng of APit) using Superscript^®^ III reverse transcriptase (Invitrogen) according to the manufacturer’s protocol. In the NHpC and PVN, TRH, TRHR1, TRHR3, UCN3, CRH2 and D2 gene expression were measured. In the APit, TSHβ, TRHR1, TRHR3, and D2 mRNA levels were determined. The primer pair for the examined genes was chosen depending upon past studies. Electrophoresis on a 3% agarose gel as well as the melting curve was utilized to identify the specificity of primers (Table 1). The PCR assay was run in triplicate for each sample and performed in 30 μl using the following conditions: one cycle at 95°C for 10 m and amplified for 40 cycles at 95°C for 30 s, 60°C for 1 m, and 72°C for 30 s. The chicken glyceraldehyde-3-phosphate dehydrogenase (GAPDH) and beta-actin (βA) were used as internal controls to normalize the mRNA levels. Relative gene expression levels of each specific gene were determined by the 2^−ΔΔCt^ method (Schmittgen and Livak, 2008). Relative expression of control groups was set to 1.

**TABLE 1:** List of primers.

Gene	Accession #	Sequences (5′-3′) (forward/reverse)	Amplicon size (bp)	References
CRH2	KU887752	CGG​AGC​AGC​GGC​AGC​GGT​AT	139	Bu et al. (2019)
		CTG​CAG​CGG​GGA​GCA​GCT​CT		
TRH	XM_025154454	CTG​GAT​GAC​ATC​CTG​CAG​AG	110	Li et al. (2020)
		CATTGTGGCAGAGGCATG		
TSHβ	XM_025143670	CCACCATCTGCGCTGGAT	128	Designed in our lab
		GCC​CGG​AAT​CAG​TGC​TGT​T		
TRH-R1	NM_204930	TGA​ATC​CCA​TCC​CTT​CGG​AC	202	Li et al. (2020)
		ACC​ACC​AGT​GTT​CGA​TAG​GG		
TRH-R3	XM_004947049	GCA​GGG​GTT​TGG​GTG​GAT​AA	163	Li et al. (2020)
		GCT​TCA​GCC​AGT​TTC​CAA​GC		
UCN3	XM_001231710	CCA​CCA​ACA​TCA​TGA​ACA​TCC​TGC​CTT​CGC​CCT​CAA​GTT​CTT	61	Ogino et al. (2014))
D2	NM_001324555	TGCGCGCGGTCAAACTT	117	Fallahshahroudi et al. (2019)
		ACT​TGG​TTC​CAC​ACT​TGC​CA		

### 2.5 Statistical analysis

Gene expression statistical analyses were conducted using JMPR pro 14.0 (SAS Institute Inc., NC). Normal distribution was first tested and thereafter differences among six independent groups for the NHpC, PVN, and APit were analyzed separately using one-way analysis of variance (ANOVA). Comparison for all pairs using Tukey’s Kramer HSD test was used to evaluate relative changes in gene expression between control and each immobilized group. Data are presented as the mean ± SEM. A probability level of *p* < 0.05 was considered statistically significant.

## 3 Results

### 3.1 Immobilization induced corticosterone increases in blood plasma

**FIGURE 1:**
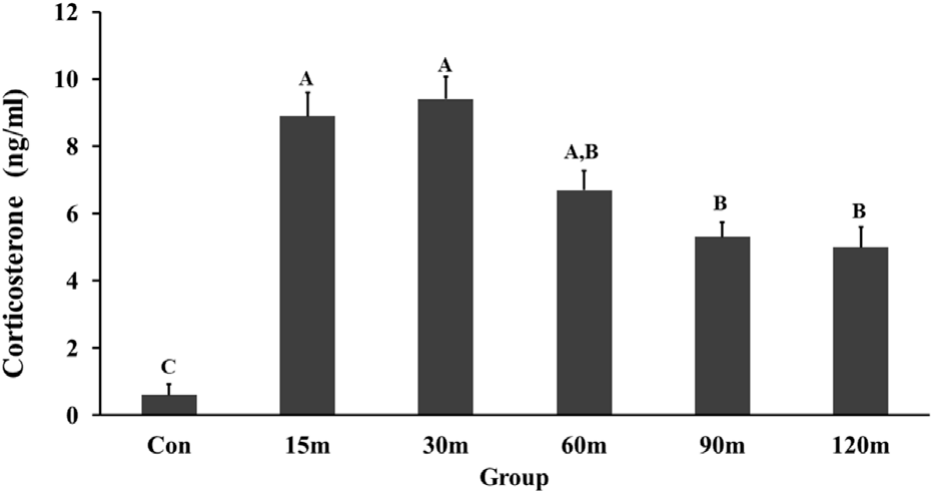
Changes in plasma corticosterone concentration in response to different times of immobilization stress. Histograms show the mean ± SEM. Significant differences (*p* < 0.05) among treatment groups are indicated by different letters above each time point. Abbreviations: Con, controls; m, minutes.

During immobilization, there was a robust and rapid increase in CORT concentration in the blood plasma (Figure 1). Specifically, the first significant increase in CORT concentration was observed at 15 m (*p* < 0.001) and peaked at 30 m (*p* < 0.001). Thereafter, CORT level declined at 60 m compared with 15 and 30 m, although it remained higher than controls until the end of all treatments. The overall significance of the change in CORT concentration between immobilized groups compared to the control group was (*p* < 0.001).

### 3.2 Gene expression data in the nucleus of the hippocampal commissure and paraventricular nucleus of the brain

#### 3.2.1 Thyrotropin-releasing hormone and its receptors

**FIGURE 2:**
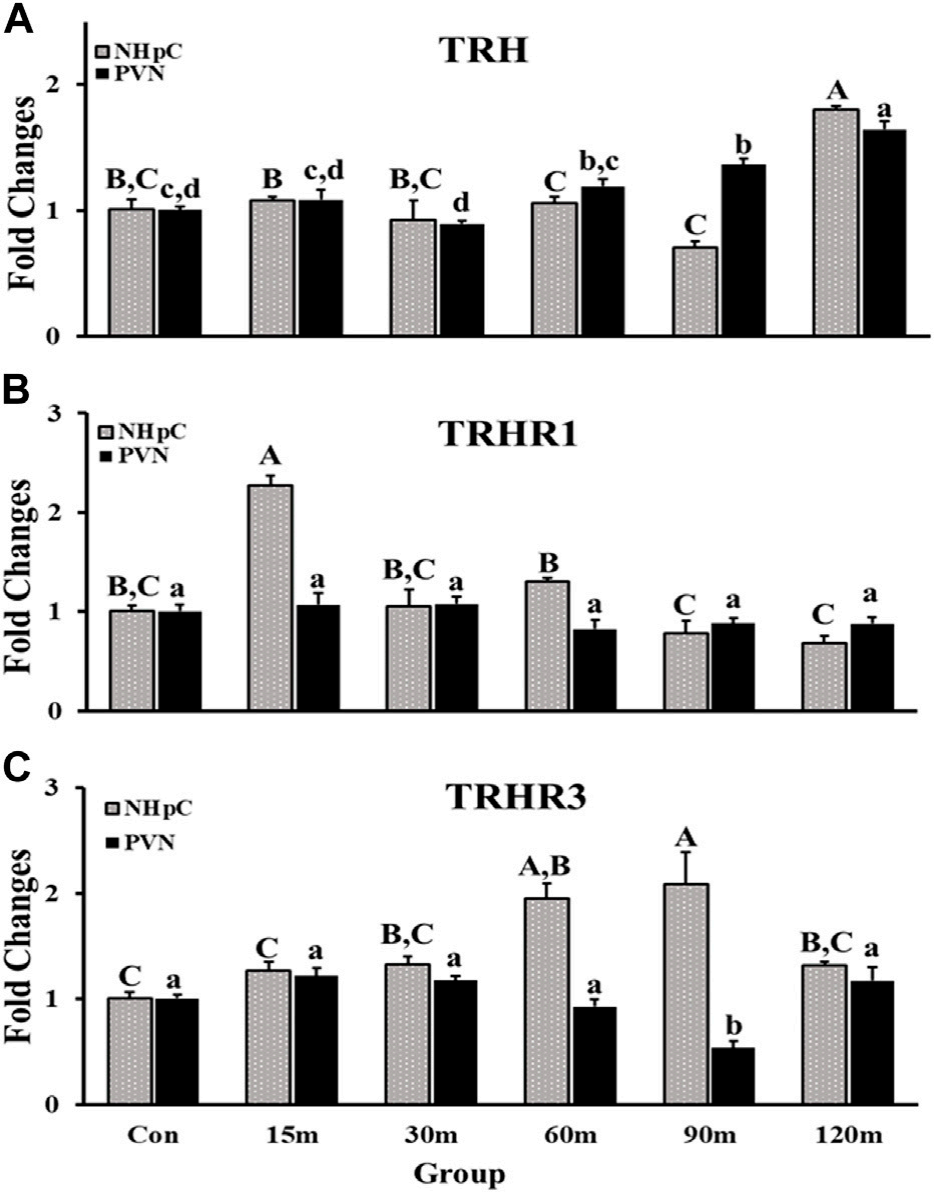
Effects of immobilization stress on relative mRNA expression levels of TRH **(A)**, TRH-R1 **(B)**, and TRH-R3 **(C)**, in the NHpC and PVN. Fold changes in relative expression levels were found using the 2^−ΔΔCt^ method after normalization with internal controls (GAPDH or *β*-Actin). Means ± SEM were determined for each gene. Significant differences (*p* < 0.05) among groups were specified by different letters above each bar or histogram. The gray box and uppercase letters refer to treatment differences among NHpC samples while the black box and lowercase letters refer to treatment differences among PVN samples.

Messenger RNA of TRH and its receptors was detected in both structures (NHpC and PVN) and showed a significant difference among treatment groups (*p* < 0.01; Figure 2A). In the NHpC, the TRH mRNA expression showed no significant differences between treatment groups compared with controls until 120 m of immobilization stress (Figure 2A). The only significant upregulation was observed at 120 m of restraint. Similarly, gene expression of TRH in the PVN did not respond until 90 m when the first significant increase was reported and peaked at 120 m of treatment (Figure 2A). In terms of receptors, a rapid, significant upregulation of TRH-R1 gene expression in the NHpC occurred at 15 m followed by a return to baseline levels throughout the treatments. While in the PVN, no changes in the mRNA expression between control and TRH-R1 treatment groups occurred (Figure 2B). Furthermore, mRNA expression of TRH-R3 in the NHpC showed a significant increase at only 60 and 90 m of immobilization stress, while TRH-R3 mRNA expression in the PVN decreased gradually, and a significant downregulation was observed at only 90 m of stress treatment before returning to the unstressed, control level at 120 m (Figure 2C).

#### 3.2.2 Type II iodothyronine deiodinase 2

**FIGURE 3:**
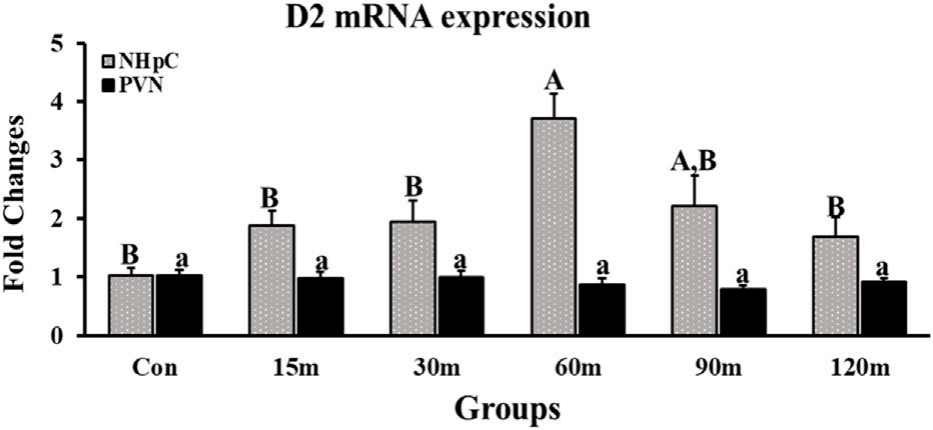
Fold changes of relative expression levels of D2 in the NHpC and PVN during immobilization stress. Data were found using the 2^−ΔΔCt^ method after normalization with internal controls (GAPDH or *β*-Actin). Means ± SEM were determined for each gene. Significant differences (*p* < 0.05) among groups were specified by different letters above each bar or histogram. The gray box and uppercase letters refer to treatment differences among NHpC samples while the black box and lowercase letters refer to treatment differences among PVN samples.

Gene expression of D2 in the NHpC was upregulated gradually, and a first significant increase was observed at only 60 m before returning to control levels at the end of treatments, 120 m (*p* < 0.01; Figure 3). In contrast to the NHpC, the PVN showed no changes throughout treatments compared with controls (*p* > 0.05).

#### 3.2.3 Corticotropin-releasing hormone 2 and urocortin 3

**FIGURE 4:**
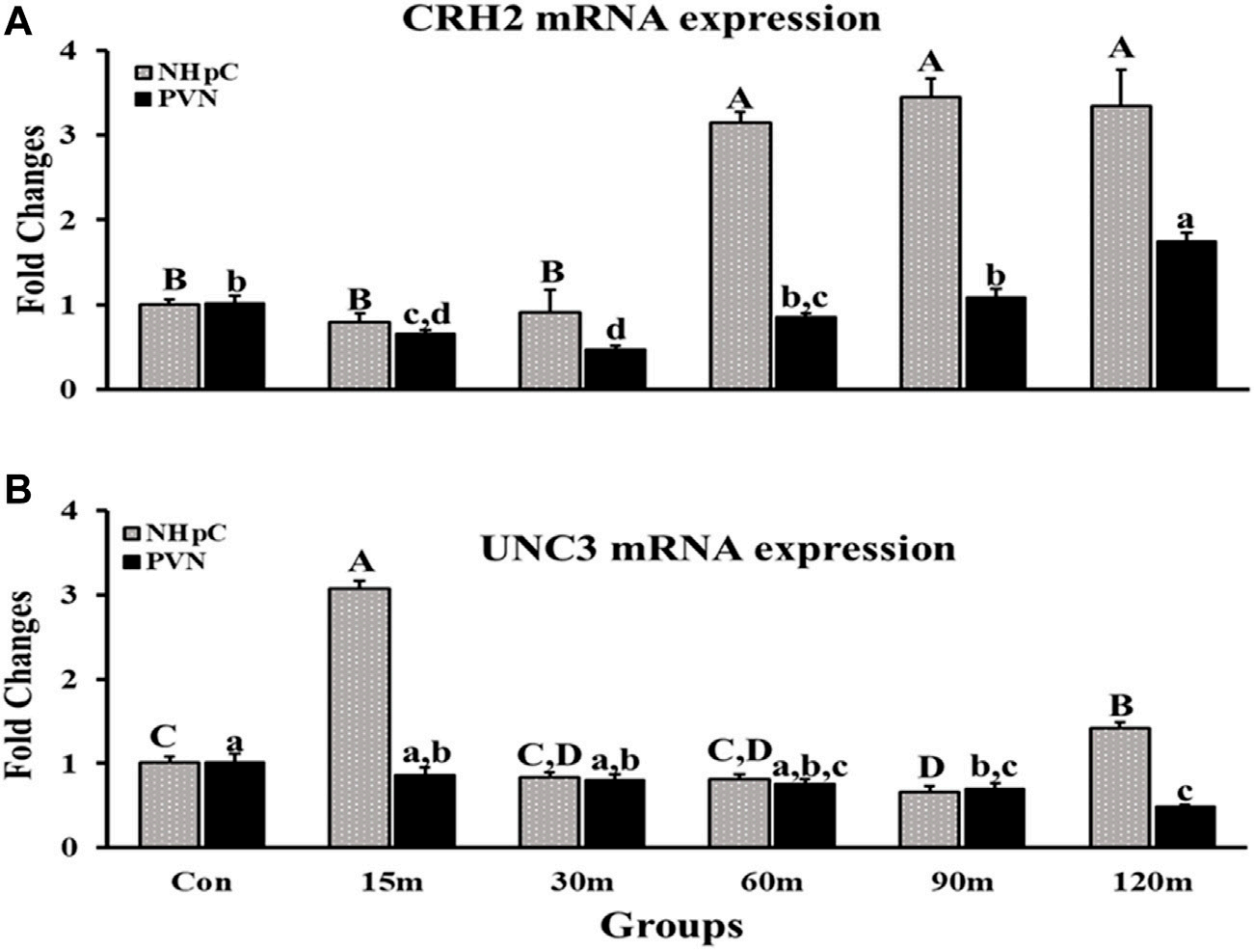
Fold changes of relative expression levels of CRH2 **(A)** and UCN3 **(B)** in the NHpC and PVN during immobilization stress. Data were found using the 2^−ΔΔCt^ method after normalization with internal controls (GAPDH or *β*-Actin). Means ± SEM were determined for each gene. Significant differences (*p* < 0.05) among groups were specified by different letters above each bar or histogram. The gray box and uppercase letters refer to treatment differences among NHpC samples while the black box and lowercase letters refer to treatment differences among PVN samples.

Corticotropin-releasing hormone 2 (CRH2) and urocortin 3 (UCN3) mRNA expression were detected in the NHpC as well as PVN and showed significant differences among treatment groups compared with controls (*p* < 0.001; Figure 4). Specifically, gene expression of CRH2 in the PVN was downregulated initially at 15 m followed by the lowest decrease at 30 m. After that, CRH2 gene expression showed a significant upregulation at 120 m, the last treatment time. In contrast, in the NHpC, a marked 3-fold increase in CRH2 mRNA occurred at 60, 90, and 120 m after IM (Figure 4A). In the NHpC, UCN3 gene expression was 3-fold higher solely at 15 m, then returned to the normal level at 30 m that lasted to 60 m. At the last sampling time, 120 m, a significant upregulation occurred (Figure 4B), however it was significantly less than the peak response of the NHpC at 15 m. In contrast, UCN3 gene expression in the PVN declined gradually and was downregulated at 90 and 120 m compared to controls (Figure 4B).

### 3.3 Gene expression data in the APit

#### 3.3.1 Thyroid stimulating hormone subunit beta, TRH-R1, and TRH-R3

**FIGURE 5:**
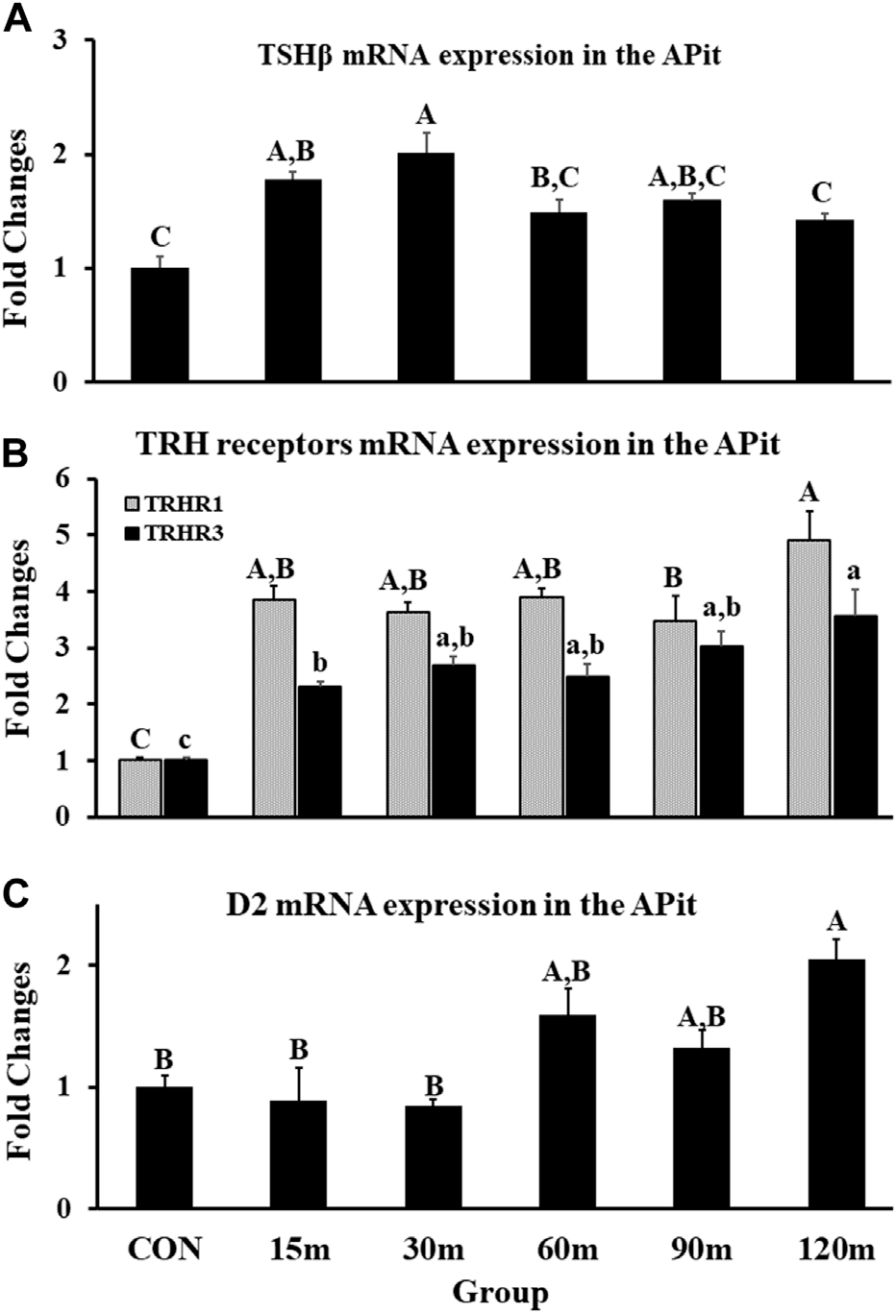
Fold changes of relative expression levels of TSHβ **(A)**, TRH receptors **(B)**, and D2 **(C)** in the APit during immobilization stress. Data were found using the 2^−ΔΔCt^ method after normalization with internal controls (GAPDH or *β*-Actin). Means ± SEM were determined for each gene. Significant differences (*p* < 0.05) among treatment groups were specified by different letters above each bar or histogram for **(A–C)**. In **(B)**, the gray box and uppercase letters show TRHR1 and the black box and lowercase letters show TRHR3 and different letters indicate significant differences among treatment groups for each gene.

Gene expression of TSHβ in the APit was measured as an indicator of thyrotrope activation during stress. A significant increase of TSHβ mRNA was documented as early as 15 m, with a peak mRNA expression at 30 m before decreasing from the peak, however continued higher than control levels to the end (*p* < 0.001; Figure 5A). Furthermore, mRNA levels of TRH receptors (TRH-R1 and TRH-R3) displayed a significant upregulation in the APit throughout treatments compared with controls. In detail, a significant increase of TRH-R1 mRNA at 15 m occurred by more than 300% that remained at the same level until 90 m. At 120 m, the peak of gene expression was documented. Additionally, TRH-R3 gene expression showed the same pattern exhibited by TRH-R1 throughout the five treatments (*p* < 0.01; Figure 5B).

#### 3.3.2 Type II iodothyronine deiodinase 2

Deiodinase 2 (D2) mRNA expression showed no changes at 15 and 30 m of IM stress compared with controls. Thereafter, the first mRNA upregulation was demonstrated at 60 m and remained at the same level at 90 m. A significant peak in D2 gene expression occurred at 120 m (*p* < 0.01; Figure 5C).

## 4 Discussion

The current study utilized a stressor, immobilization, to examine changes in gene expression of a panel of genes related to the thyroid axis occurring in the brain and APit. A goal was to determine their possible roles in the avian neuroendocrine regulation of stress. What is unknown is the timing of neural and endocrine components of the HPT axis compared with the HPA axis utilizing the same stressor. Therefore, we compared the timing of significant changes in gene expression within the same brain structures (NHpC and PVN), TSHβ in the APit, and the production of the stress hormone, CORT. If the timing of neural and APit components of the HPT axis occurs within the HPA axis time frame, then there would be evidence to consider components of the HPT axis as complementary to those of the HPA axis regulating stress.

### 4.1 Role of neural secretion from nucleus of the hippocampal commissure and paraventricular nucleus in the increase of corticosterone concentration in blood and thyroid stimulating hormone subunit beta mRNA levels in the anterior pituitary

The sequence of gene activation within the traditional HPA axis of birds has been completed with two different stressors in our lab. Utilizing feed deprivation (Nagarajan et al., 2017b; Kadhim et al., 2019) or immobilization (IM, Kadhim et al., 2021) suggested that the avian HPA axis may include an additional structure, the NHpC, located in the septal region just dorsal to the hypothalamus. Regardless of the stressor, the sequence of gene activation for maintaining the CORT response was: 1) NHpC (CRH), 2) PVN (CRH), and 3) PVN (arginine vasotocin, AVT). An intriguing result of our recent stress study using IM stress was the robust, increased response of CRH-R2 in the APit (Kadhim et al., 2021). It alerted us that in birds, CRH-R2 was located in thyrotropes, not corticotropes (De Groef et al., 2003). Hence, there may be components of the HPT axis that may play a role in the avian stress response. If so, the APit would need to synthesize both POMC, the pre-pro hormone that produces adrenocorticotropic hormone (ACTH) and TSH following the same stressor. If both the HPA and HPT axes are activated concurrently following a stressor and the NHpC likewise displays components of the HPT axis activated during sampling times, would it provide further evidence to include the NHpC in the avian stress pathway?

In the current study, IM stress induced a significant increase in CORT concentration in the blood plasma that started at 15 m of IM stress, peaked at 30 m, and remained higher than control levels to the end of treatments. Similarly, the major component of the HPT axis, TSHβ, showed a significant increase in mRNA at 15 m that continued to the end of treatments. The upregulation of TSHβ gene in the APit could be mediated *via* the action of CRH-like peptides secreted from the NHpC and PVN activating CRH-R2 located on thyrotropes. Our past HPA data showed rapid upregulation in CRH mRNA in the NHpC and PVN followed by a significant increase of hn/mRNA POMC in the APit. Interestingly, CRH-R2 displayed a significant increase in gene expression in the APit in all immobilized groups (Kadhim et al., 2021). In summary, the significant increase of TSHβ, a component of the HPT axis matched a similar timing of a significant increase in POMC, a component of the HPA axis (Kadhim et al., 2021). The possible contribution of a neural structure contributing to the activation of the HPT axis is covered next.

### 4.2 Role of thyrotropin-releasing hormone, TRH-R1, and TRH-R3 in the neuroendocrine regulation of stress

A curious result in the study was the significant increase in gene expression of TRH-R1 within the NHpC at 15 m. Additionally, a significant upregulation of TRH-R3 mRNA expression in the NHpC occurred at 60 m near the end of treatment groups. A main issue with TRH-R1 is that one would expect a possible change in expression of TRH within the NHpC, however, no change was observed from 15 to 90 m in that structure. A possible explanation is that the NHpC is surrounded by three circumventricular organs, the organum vasculosum of the lamina terminalis (OVLT), sub-septal organ (SSO), and choroid plexus (CP). The OVLT and SSO reside in the anterior and dorsal region of the third ventricle at its midline while the CP occupies the base of the two lateral ventricles. The NHpC is located at the midline, just dorsal to the third ventricle. A study has shown that the chicken choroid plexus contains type II iodothyronine deiodinase (D2) and produces T_3_ (Verhoelst et al., 2004). The epithelial cells lining the walls of the avian lateral ventricles and epithelial cells of the CP contain the D2 protein. Hence, within the avian brain, T_3_ can originate in non-neuronal cells in the CP (Verhoelst et al., 2004). Therefore, it is possible that a local source of T_3_ was provided by the CP during that stressful 15 m period as evidenced by the significant upregulation of the TRH-R1 in the NHpC (Figure 2B). This suggests that a change in gene expression for TRH is not required to provide the needed source of energy *via* the classical HPT axis.

Our current study shows that both the NHpC and PVN dissected structures likewise contain D2 mRNA. Note that the NHpC produced some increases in D2 mRNA at 15 and 30 m and showed nearly a 4-fold increase in gene expression from controls at 60 m following IM stress. Currently, we do not know whether the functional gene, D2, occurs in glia or neurons in the NHpC and PVN. Birds appear to have at least three sources within the brain for the production of T_3_: 1) the traditional HPT axis, 2) the choroid plexus of the brain (Verhoelst et al., 2004) and 3) the presence of the D2 gene in individual brain structures, such as the NHpC and PVN (current study).

### 4.3 Role of corticotropin-releasing hormone and urocortin3 in upregulation of thyroid stimulating hormone gene *via* CRH-R2 during immobilization stress

Our data, for the first time, documented changes in CRH2 mRNA following IM stress occurring in the NHpC and PVN. Specifically, gene expression significantly increased 3-fold at 60, 90, and 120 m in the NHpC and significantly increased in the PVN at the last sampling point, 120 m. Note that gene expression for CRH2 displayed an extended delay of 1 h following IM stress before a significant increase was first detected in the NHpC. Gene expression in the PVN showed a significant decline at 15 and 30 m and returned to baseline at the 60 and 90 m sampling times. A significant increase in CRH2 mRNA was demonstrated solely during the last sampling point of 120 m. Overall, the response of the avian CRH2 gene to IM stress appears to be dependent upon the neural structure, was totally different between the hypothalamic PVN and the extra-hypothalamic NHpC and in both structures displayed a late, positive response to IM stress. Since CRH2 has a high affinity for CRH-R2 located on thyrotropes (Bu et al., 2019), its upregulation appears to sustain thyrotrope activation as measured by TSHβ mRNA levels in the APit.

Besides CRH and CRH2, another peptide urocortin 3 (UCN3) displayed a highly significant 3-fold increase in gene expression within the NHpC at 15 m. Past studies in mammals showed the peptide UCN (Vaughan et al., 1995) and specifically UCN3, within the family of CRH-like peptides, has a high affinity for CRH-R2 (Lewis et al., 2001). Thereby UCN3 may contribute to the HPT axis. Past studies, using human UCN3 (hUCN3, stresscopin), showed that hUCN3 was effective in releasing chicken TSH; however, was less effective than CRH (De Groef et al., 2003). The chicken UCN3 has been cloned and its possible function/s have been suggested (Grommen et al., 2017). Is there any evidence that the significant increase in UCN3 mRNA in the NHpC at 15 m following immobilization could rapidly affect APit production of TSH? This would require a direct or efficient neural connection between the NHpC and median eminence resulting in a fast release of the peptide into the portal capillary system for transport to the APit. A past behavioral study using mature broiler male-male and male-female interactions provides evidence. In the experiment, four groups were designed. In all experimental groups, the fixed brains were sectioned and used for visualization of FOSir cells. Results showed that all treatments revealed intense immunoreactive (ir) cells in the dorsal region of the NHpC where CRH neurons are located. The greatest ir occurred with the male-male interactions (Xie et al., 2010). Hence that male-male interaction as well as the other three male-female interactions resulted in activating the HPA axis *via* increased activity of neurons known to help initiate the ultimate production of the stress hormone, CORT.

In the current study, the peak response of UCN3 mRNA occurred at 15 m in the NHpC and likewise a significant increase in TSHβ was documented in the APit at 15 m. We, therefore, suggest that UCN3 neurons in the NHpC have contributed to that significant increase in APit TSHβ at 15 m and thereafter. We have no immunocytochemical data showing that UCN3 neurons exist in the NHpC. However, the dramatic gene expression changes shown in the NHpC of UCN3 provide strong evidence of their existence, particularly contributing to the early significant increase in TSHβ within the APit. Similarly, we have no direct evidence that UCN3 neurons in the NHpC project to the ME. However, there exists an alternative possibility. The NHpC resides at the midline just dorsal to the hypothalamus and sits directly above the third ventricle (VIII). Cells within the NHpC could have axons projecting to the ME or directly beneath the NHpC into the VIII containing cerebrospinal fluid. Therefore, NHpC secretions of UNC3 into the VIII would eventually be transported to the ME.

### 4.4 Role of the HPT axis in the neuroendocrine regulation of stress

Data collected in the current experiments suggest that genes associated with the major components of the HPT axis should be seriously examined in future studies involving the avian HPA axis and the neuroendocrine stress response. There appear to be at least three neuropeptide genes in the HPT axis impacting the neuroendocrine regulation of stress: TRH, CRH2, and UCN3. Additionally, the APit hormone gene TSHβ, two receptors, TRH-R1, TRH-R3 and enzyme gene D2 likewise play a role. Importantly, all the previously named genes were activated within the time span when genes associated with the avian HPA axis displayed clear changes in gene expression following activation of the same stressor. The following summarizes the timing of some of the seven HPT genes and their possible role in interacting with the HPA axis. Specifically, a significant, delayed increase in gene expression of TRH occurred in the PVN at 90 and 120 m following IM stress, while a highly significant increase in TRH mRNA occurred in the NHpC at 120 m. A second distinct gene, CRH2, showed a highly significant increase of its mRNA at 60, 90, and 120 m in the NHpC while the PVN showed a delayed activation at 120 m.

The third gene listed above as part of the avian HPT axis was UCN3. This gene displayed activation early and late in the NHpC, specifically at only the first (15 m) and last sampling points (120 m) following IM stress. Gene expression of UCN3 was six times greater at 15 m compared to its significant increase at 120 m. UCN3 was shown to bind to CRH-R2 and failed to activate CRH-R1 even at high concentrations. Similarly, Hsu and Hsueh (2001) reported that human UCN3 or stresscopin did not stimulate ACTH in cell culture studies nor *in vivo* (Hsu and Hsueh 2001). In chickens, human UCN3 was shown to stimulate TSH release in APit glands (De Groef et al., 2003). The large increase at 15 m suggests another possible function of UCN3, perhaps related to a stress behavioral response at the level of the brain. In an open field behavioral test in rodents or a dark-light emergence test, mice injected with 20 ng UCN3 intracerebroventricularly (icv) showed significantly less anxiety-like behavior than controls that was associated with no changes in plasma concentrations of ACTH and CORT (Venihaki et al., 2004). However, mice lacking the CRH-R2 were shown hypersensitive to stress and displayed anxiety-like behavior (Bale et al., 2000). Hence, in mammals, UCN3 is associated with anxiety-like behavior. Our UCN3 data suggest a possible behavioral role of UCN3 in birds related to anxiety. Our stress protocol involved our picking up each bird, wrapping it in a harness so that it could not move its wings nor stand up, then quickly returning the bird to its cage. Thus, it is likely that the protocol we utilized for immobilizing each bird was initially quite stressful and UCN3 may have enabled coping with that restraint. Additionally, the delayed, significantly increased UCN3 mRNA at 120 m appears to be a natural response of the NHpC to activate the HPT axis to generate the needed energy to cope with the continued stressor IM.

Activation of the three genes (TRH, CRH2, and UNC3) is reflected in the sustained, continued significant upregulation of TSHβ gene expression and increase of TRH-R1 and -R3 mRNA in the APit, while the significant increase of D2 at 120 m in the APit appears to generate T_3_ from T_4_ (Figure 5). Hence, three genes acting within two neural structures associated with the HPA axis appear to likewise activate TSHβ, the critical APit hormone of the HPT axis, to produce the necessary energy for providing a complementary means to address IM stress. Overall, concurrent activation of both the HPA and HPT axes appears to be an essential, physiological response in an attempt to maintain homeostasis in birds undergoing a physiological and/or mental challenge.

## 5 Summary

The study addressed a group of genes associated with the HPT axis to ascertain their change in gene expression following IM stress. Genes in two distinct brain structures and regions (NHpC in the septum and PVN in the hypothalamus) and the APit were examined to compare them with activation of genes regarded as components of the avian HPA axis. The objective was to determine whether genes of the HPT axis may clarify our understanding of the neuroendocrine regulation of stress in birds. Results showed that the timing of the pattern of expression of the major gene, TSHβ of the HPT axis, at the level of the APit, matched well with the increase in CORT which is the final product of HPA axis activation. At the neural level, two peptides in the NHpC responded rapidly to IM stress. Both CRH and UNC3 showed significantly increased mRNA at 15 m, contributing to activation of the HPT axis, as each is known to bind to CRH-R2. In both the NHpC and PVN, CRH2 and TRH responded late to IM with significant increases in mRNA, thereby sustaining activation of the HPT axis. Overall, the timing of significant changes in APit and neural components of the HPT axis following IM stress occurred within the time frame of previous data addressing the HPA axis. Additionally, current data show that the NHpC is a major player in both avian HPT and HPA axes, supporting the proposal that the structure should be included in the neuroendocrine regulation of stress in birds.

## Data Availability

The original contributions presented in the study are included in the article/supplementary material, further inquiries can be directed to the corresponding author.
